# {2-[*tert*-But­yl(pyridin-2-yl)phosphino]phen­yl}diphenyl­phosphine

**DOI:** 10.1107/S2414314626007844

**Published:** 2026-08-04

**Authors:** Joseph Görl, Rauf Razzaq, Anke Spannenberg, Ralf Jackstell

**Affiliations:** a RWTH Aachen University, Institute of Technical and Macromolecular Chemistry, Worringerweg 2, 52074 Aachen, Germany; b Leibniz Institute for Catalysis, Albert-Einstein-Str. 29a, 18059 Rostock, Germany; University of Aberdeen, United Kingdom

**Keywords:** crystal structure, phosphine ligand, P,P,N donor

## Abstract

The title compound, C_27_H_27_NP_2_, is a new potential asymmetric P,P,N ligand and consists of a di­phenyl­phosphino and a *tert*-but­yl(pyridin-2-­yl)phosphino group bridged by a 1,2-phenyl­ene backbone. The dihedral angles between the central and pendant aromatic rings are 83.07 (6), 85.99 (5) and 82.80 (6)°. In the extended structure, weak C—H⋯π inter­actions link the mol­ecules.

## Abstract 2

The title compound, C_27_H_27_NP_2_, consists of a di­phenyl­phosphino and a *tert*-but­yl(2-pyrid­yl)phosphino group bridged by a 1,2-phenyl­ene backbone.

## Graphical abstract

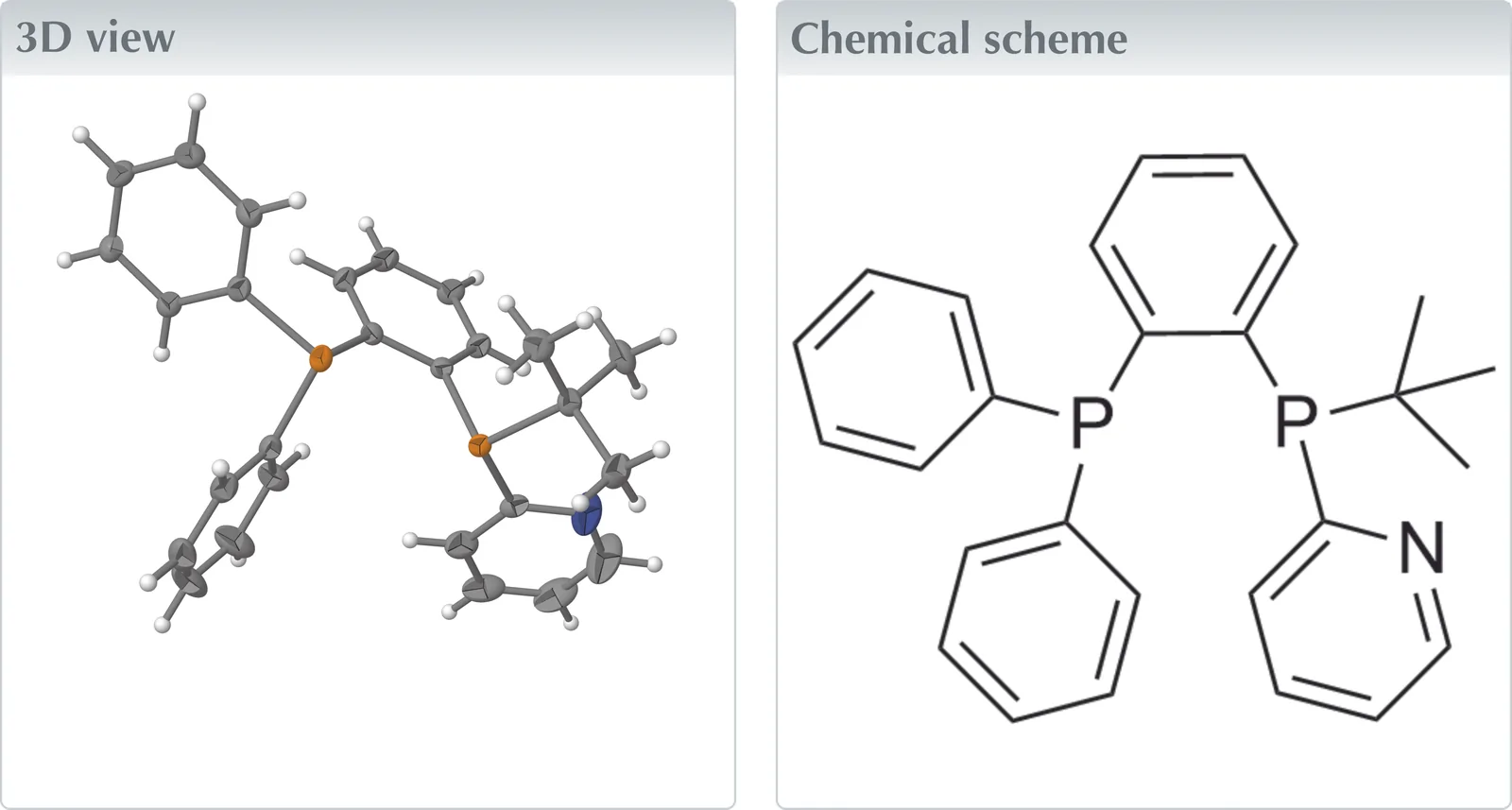

## Structure description

**Figure 1:**
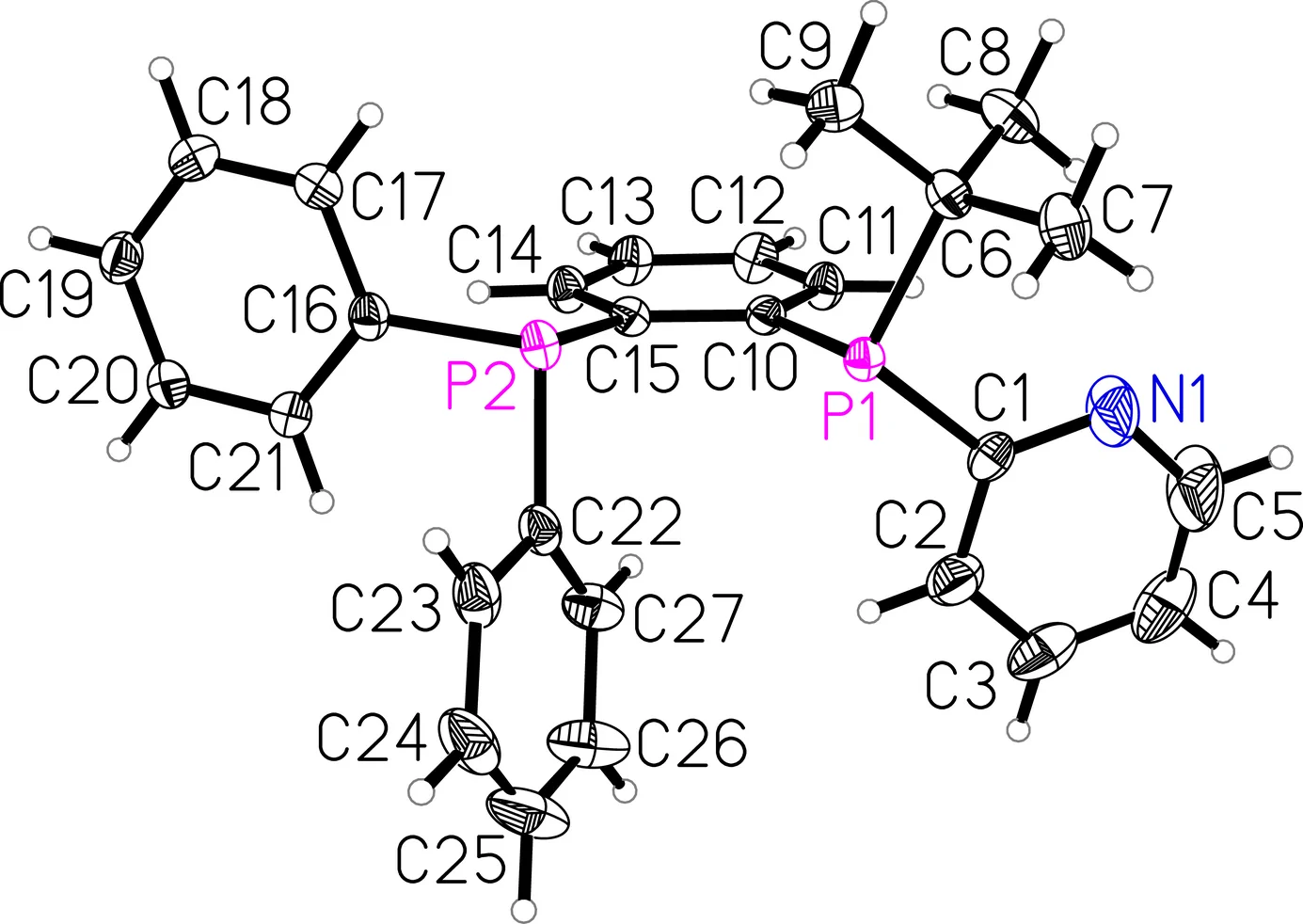
The mol­ecular structure of the title compound. Displacement ellipsoids correspond to the 50% probability level.

The title compound, C_27_H_27_NP_2_, consists of di­phenyl­phosphino and *tert*-but­yl(pyridin-2-yl)phosphino groups bridged by a 1,2-phenyl­ene backbone (Fig. 1 ▸). This compound can act as a tridentate P,P,N donor ligand containing two phosphine donors and one pyridyl nitro­gen donor. This opens new possibilities for multi-functional ligand and complex design for various chemical transformations in homogeneous catalysis (Breit, 2000 ▸; Fablet *et al.*, 2024 ▸) where the steric and electronic properties of the phospho­rus ligands strongly influence catalyst performance. In the crystal structure, the steric demand results from the presence of a phenyl­ene backbone. The dihedral angles between the central C10–C15 ring and pendant C1–C5/N1, C16–C21 and C22–C27 rings are 83.07 (6), 85.99 (5) and 82.80 (6)°, respectively. The N1—C1—P1—C10 torsion angle is 108.10 (11)° and two short intra­molecular C—H⋯N contacts (Table 1 ▸) occur. In the extended structure, some weak C—H⋯π inter­actions are observed.

## Synthesis and crystallization

The title compound was obtained by dissolving (2-bromo­phen­yl)(*tert*-but­yl)(pyridin-2-yl)phosphine (6.02 g, 18.8 mmol) in 70 ml of tetra­hydro­furan (THF) and cooled to 203 K. A 1.6 *N* solution of *n*-butyl­lithium in THF (11.75 ml, 18.8 mmol) was added dropwise without raising the inter­nal temperature of the solution above 213 K. The temperature was maintained, and the reaction mixture was stirred for 45 min. Chloro­diphenyl­phosphine (4.14 g, 3.46 ml, 18.8 mmol) was added dropwise. The cooling was removed and upon reaching room temperature, the solution was stirred for further 15 min. The solvent was removed *in vacuo*, leaving behind a brown solid, which was redissolved in a mixture of 40 ml of di­ethyl­ether and 30 ml of toluene. The solution was washed with 20 ml of distilled water three times and the solvent was removed *in vacuo*. The resulting solid was crystallized from acetone solution, giving 3.83 g (8.97 mmol, 47.7%) of the product as a crystalline material.

^1^H-NMR (300 MHz, CD_2_Cl_2_): δ = 8.61–8.64 (*m*, 1 H, H6), 7.66–7.72 (*m*, 1 H, H4), 7.22–7.40 (*m*, 13 H, H5, H10, H11, H15–H17) 7.03–7.08 (*m*, 1 H, H3), 6.95–7.01 (*m*, 1 H, H9), 6.89–6.95 (*m*, 1 H, H12), 1.33 (*d*, ^3^*J*_HP_ = 12.7 Hz, 9 H, 1H) ppm. ^13^C{^1^H}-NMR (300 MHz, CD_2_Cl_2_): δ = 165.40 (*dd*, ^1^*J*_CP_ = 11.8 Hz, ^4^*J*_CP_ = 7.4 Hz, 1 C, C7), 149.18 (*d*, ^3^*J*_CP_ = 5.5 Hz, 1 C, C6), 146.88 (*dd*, ^1^*J*_CP_ = 35.3 Hz, ^2^*J*_CP_ = 11.2 Hz, 1 C, C8), 142.00 (*dd*, ^1^*J*_CP_ = 29.3 Hz, ^2^*J*_CP_ = 15.3 Hz, 1 C, C13), 137.84–138.51 (*m*, 2 C, C4, C12), 136.35 (*dd*, ^1^*J*_CP_ = 4.9 Hz, ^4^*J*_CP_ = 2.7 Hz, 2 C, C14), 134.47 (*d*, ^2^*J*_CP_ = 6.3 Hz, 1 C, C9), 134. 38 (*d*, ^2^*J*_CP_ = 20.4 Hz, 4 C, C15), 133.76 (*d*, ^3^*J*_CP_ = 19.2 Hz, 4 C, C16), 133.29 (*d*, ^4^*J*_CP_ = 9.1 Hz, 2 C, C17) 129.62 (*d*, ^3^*J*_CP_ = 1.6 Hz, 1 C, C10), 129.02 (*d*, ^3^*J*_CP_ = 29.4 Hz, 1 C, C11), 128.23–128.57 (*m*, 3 C, C3, C4, C5), 33.20 (*dd*, ^1^*J*_CP_ = 15.7 Hz, ^4^*J*_CP_ = 2.9 Hz, 1 C, C2), 28.37 (*dd*, ^2^*J*_CP_ = 12.9 Hz, ^5^*J*_CP_ = 2.0 Hz, 3 C, C1) ppm. ^31^P{^1^H}-NMR (300 MHz, CD_2_Cl_2_): δ = 3.06 (*d*, ^3^*J*_PP_ = 154 Hz, 1 P, *R*-PPh_2_), −9.90 (*d*, ^3^*J*_PP_ = 154 Hz, 1 P, *R*—P(*tert*-but­yl)(pyrid­yl) ppm.

## Refinement

Crystal data, data collection and structure refinement details are summarized in Table 2 ▸.

## Supplementary Material

Crystal structure: contains datablock(s) I. DOI: 10.1107/S2414314626007844/hb4567sup1.cif

Structure factors: contains datablock(s) I. DOI: 10.1107/S2414314626007844/hb4567Isup2.hkl

Supporting information file. DOI: 10.1107/S2414314626007844/hb4567Isup3.cml

CCDC reference: 2578570

Additional supporting information:  crystallographic information; 3D view; checkCIF report

## Figures and Tables

**Table 1:** C—H⋯N contacts and C—H⋯π interactions (Å, °) *Cg*2, *Cg*3 and *Cg*4 are the centroids of the C10–C15, C16–C21 and C22–C27 rings, respectively.

*D*—H⋯*A*	*D*—H	H⋯*A*	*D*⋯*A*	*D*—H⋯*A*
C7—H7*A*⋯N1	0.98	2.51	3.1946 (18)	127
C8—H8*B*⋯N1	0.98	2.48	3.1921 (19)	129
C9—H9*B*⋯*Cg*3^i^	0.98	2.97	3.8163 (14)	145
C13—H13⋯*Cg*4^ii^	0.95	2.85	3.6835 (12)	146
C14—H14⋯*Cg*3	0.95	2.97	3.7522 (12)	140
C18—H18⋯*Cg*2^iii^	0.95	2.71	3.6314 (12)	163

Symmetry codes: (i) 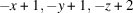; (ii) 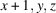; (iii) 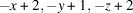.

**Table 2:** Experimental details

Crystal data	
Chemical formula	C_27_H_27_NP_2_
*M*_r_	427.43
Crystal system, space group	Triclinic, *P* 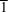
Temperature (K)	150
*a*, *b*, *c* (Å)	7.8716 (7), 11.1132 (9), 13.3896 (11)
α, β, γ (°)	91.8079 (18), 100.1759 (17), 97.1105 (17)
*V* (Å^3^)	1142.30 (17)
*Z*	2
Radiation type	Mo *K*α
μ (mm^−1^)	0.20
Crystal size (mm)	0.36 × 0.30 × 0.20

Data collection	
Diffractometer	Bruker APEXII CCD
Absorption correction	Multi-scan (*SADABS*; Krause *et al.*, 2015 ▸)
*T*_min_, *T*_max_	0.93, 0.96
No. of measured, independent and observed [*I* > 2σ(*I*)] reflections	67091, 5960, 5557
*R*_int_	0.020
(sin θ/λ)_max_ (Å^−1^)	0.678

Refinement	
*R*[*F*^2^ > 2σ(*F*^2^)], *wR*(*F*^2^), *S*	0.030, 0.084, 1.05
No. of reflections	5960
No. of parameters	274
H-atom treatment	H-atom parameters constrained
Δρ_max_, Δρ_min_ (e Å^−3^)	0.36, −0.22

Computer programs: *APEX2* (Bruker, 2014 ▸) and *SAINT* (Bruker, 2013 ▸), *SHELXT* (Sheldrick, 2015*a* ▸), *SHELXL* (Sheldrick, 2015*b* ▸), *XP* in *SHELXTL* (Sheldrick, 2008 ▸) and *publCIF* (Westrip, 2010 ▸).

## full crystallographic data

### {2-[tert-Butyl(pyridin-2-yl)phosphino]phenyl}diphenylphosphine Crystal data

**Table:**

C_27_H_27_NP_2_	*Z* = 2
*M**_r_* = 427.43	*F*(000) = 452
Triclinic, *P*1	*D*_x_ = 1.243 Mg m^−^^3^
*a* = 7.8716 (7) Å	Mo *K*α radiation, λ = 0.71073 Å
*b* = 11.1132 (9) Å	Cell parameters from 9606 reflections
*c* = 13.3896 (11) Å	θ = 2.4–28.8°
α = 91.8079 (18)°	µ = 0.20 mm^−^^1^
β = 100.1759 (17)°	*T* = 150 K
γ = 97.1105 (17)°	Block, colourless
*V* = 1142.30 (17) Å^3^	0.36 × 0.30 × 0.20 mm

### {2-[tert-Butyl(pyridin-2-yl)phosphino]phenyl}diphenylphosphine Data collection

**Table:**

Bruker APEXII CCD diffractometer	5960 independent reflections
Radiation source: fine-focus sealed tube	5557 reflections with *I* > 2σ(*I*)
Detector resolution: 8.3333 pixels mm^-1^	*R*_int_ = 0.020
φ and ω scans	θ_max_ = 28.8°, θ_min_ = 1.6°
Absorption correction: multi-scan (SADABS; Krause *et al.*, 2015)	*h* = −10→10
*T*_min_ = 0.93, *T*_max_ = 0.96	*k* = −15→15
67091 measured reflections	*l* = −18→18

### {2-[tert-Butyl(pyridin-2-yl)phosphino]phenyl}diphenylphosphine Refinement

**Table:**

Refinement on *F*^2^	0 restraints
Least-squares matrix: full	Hydrogen site location: inferred from neighbouring sites
*R*[*F*^2^ > 2σ(*F*^2^)] = 0.030	H-atom parameters constrained
*wR*(*F*^2^) = 0.084	*w* = 1/[σ^2^(*F*_o_^2^) + (0.0428*P*)^2^ + 0.4017*P*] where *P* = (*F*_o_^2^ + 2*F*_c_^2^)/3
*S* = 1.05	(Δ/σ)_max_ = 0.001
5960 reflections	Δρ_max_ = 0.36 e Å^−^^3^
274 parameters	Δρ_min_ = −0.22 e Å^−^^3^

### {2-[tert-Butyl(pyridin-2-yl)phosphino]phenyl}diphenylphosphine Special details

**Table:**

Geometry. All esds (except the esd in the dihedral angle between two l.s. planes) are estimated using the full covariance matrix. The cell esds are taken into account individually in the estimation of esds in distances, angles and torsion angles; correlations between esds in cell parameters are only used when they are defined by crystal symmetry. An approximate (isotropic) treatment of cell esds is used for estimating esds involving l.s. planes.

### {2-[tert-Butyl(pyridin-2-yl)phosphino]phenyl}diphenylphosphine Fractional atomic coordinates and isotropic or equivalent isotropic displacement parameters (Å^2^)

**Table:**

	*x*	*y*	*z*	*U*_iso_*/*U*_eq_	
C1	0.32534 (12)	0.18587 (10)	0.60893 (8)	0.02296 (19)	
C2	0.28865 (16)	0.26468 (12)	0.53202 (9)	0.0355 (3)	
H2	0.305204	0.349900	0.547051	0.043*	
C3	0.22765 (17)	0.21822 (15)	0.43302 (10)	0.0435 (3)	
H3	0.205756	0.271065	0.379167	0.052*	
C4	0.1995 (2)	0.09449 (16)	0.41425 (10)	0.0505 (4)	
H4	0.157672	0.059450	0.347438	0.061*	
C5	0.2336 (3)	0.02348 (16)	0.49509 (12)	0.0643 (5)	
H5	0.211051	−0.062102	0.482346	0.077*	
C6	0.42574 (13)	0.12441 (9)	0.82445 (8)	0.02225 (19)	
C7	0.24175 (15)	0.05800 (11)	0.82173 (10)	0.0322 (2)	
H7A	0.198835	0.015683	0.755028	0.048*	
H7B	0.163842	0.117023	0.833796	0.048*	
H7C	0.245172	−0.001085	0.874618	0.048*	
C8	0.54692 (16)	0.03401 (10)	0.80124 (10)	0.0321 (2)	
H8A	0.664946	0.076579	0.805694	0.048*	
H8B	0.505166	−0.003202	0.732548	0.048*	
H8C	0.548296	−0.029296	0.850588	0.048*	
C9	0.49409 (16)	0.18470 (11)	0.93116 (8)	0.0306 (2)	
H9A	0.503670	0.122008	0.980971	0.046*	
H9B	0.413300	0.239414	0.947727	0.046*	
H9C	0.608924	0.231152	0.932890	0.046*	
C10	0.62729 (12)	0.31172 (8)	0.72764 (7)	0.01762 (17)	
C11	0.72339 (13)	0.24493 (9)	0.67294 (8)	0.02238 (19)	
H11	0.671746	0.168114	0.641456	0.027*	
C12	0.89277 (13)	0.28873 (10)	0.66378 (8)	0.0257 (2)	
H12	0.957337	0.241007	0.628230	0.031*	
C13	0.96706 (13)	0.40239 (10)	0.70676 (8)	0.0255 (2)	
H13	1.082170	0.433398	0.699877	0.031*	
C14	0.87267 (13)	0.47082 (9)	0.75992 (8)	0.02235 (19)	
H14	0.923905	0.549081	0.788404	0.027*	
C15	0.70346 (12)	0.42681 (8)	0.77241 (7)	0.01798 (17)	
C16	0.74811 (13)	0.63927 (9)	0.90193 (7)	0.02054 (18)	
C17	0.86099 (14)	0.62644 (9)	0.99293 (8)	0.0260 (2)	
H17	0.847739	0.553460	1.027483	0.031*	
C18	0.99244 (15)	0.71938 (10)	1.03341 (8)	0.0292 (2)	
H18	1.069724	0.709009	1.094633	0.035*	
C19	1.01101 (14)	0.82715 (10)	0.98463 (8)	0.0267 (2)	
H19	1.101408	0.890384	1.012042	0.032*	
C20	0.89724 (13)	0.84230 (9)	0.89577 (8)	0.0243 (2)	
H20	0.908366	0.916664	0.862933	0.029*	
C21	0.76681 (13)	0.74904 (9)	0.85447 (8)	0.02204 (18)	
H21	0.689654	0.760101	0.793373	0.026*	
C22	0.43871 (12)	0.58380 (9)	0.75901 (8)	0.02325 (19)	
C23	0.32287 (14)	0.65208 (10)	0.79593 (10)	0.0318 (2)	
H23	0.320156	0.656429	0.866595	0.038*	
C24	0.21165 (16)	0.71362 (11)	0.72955 (13)	0.0421 (3)	
H24	0.135332	0.761420	0.755295	0.051*	
C25	0.21165 (18)	0.70561 (14)	0.62686 (14)	0.0490 (4)	
H25	0.134293	0.746912	0.581786	0.059*	
C26	0.32416 (19)	0.63748 (15)	0.58911 (12)	0.0466 (3)	
H26	0.323595	0.631456	0.518116	0.056*	
C27	0.43830 (15)	0.57773 (11)	0.65544 (9)	0.0314 (2)	
H27	0.516850	0.532247	0.629354	0.038*	
N1	0.2969 (2)	0.06600 (11)	0.59120 (9)	0.0481 (3)	
P1	0.40197 (3)	0.25420 (2)	0.73905 (2)	0.01760 (7)	
P2	0.58338 (3)	0.50958 (2)	0.85282 (2)	0.02000 (7)	

### {2-[tert-Butyl(pyridin-2-yl)phosphino]phenyl}diphenylphosphine Atomic displacement parameters (Å^2^)

**Table:**

	*U*^11^	*U*^22^	*U*^33^	*U*^12^	*U*^13^	*U*^23^
C1	0.0170 (4)	0.0292 (5)	0.0219 (4)	0.0007 (3)	0.0037 (3)	−0.0027 (4)
C2	0.0340 (6)	0.0415 (6)	0.0275 (5)	0.0010 (5)	−0.0010 (4)	0.0031 (5)
C3	0.0338 (6)	0.0691 (9)	0.0245 (5)	0.0012 (6)	0.0005 (5)	0.0047 (6)
C4	0.0488 (8)	0.0720 (10)	0.0246 (6)	−0.0073 (7)	0.0038 (5)	−0.0143 (6)
C5	0.1049 (15)	0.0445 (8)	0.0351 (7)	−0.0106 (9)	0.0079 (8)	−0.0178 (6)
C6	0.0239 (4)	0.0193 (4)	0.0244 (4)	0.0020 (3)	0.0070 (4)	0.0030 (3)
C7	0.0307 (5)	0.0271 (5)	0.0391 (6)	−0.0048 (4)	0.0125 (5)	0.0038 (4)
C8	0.0374 (6)	0.0245 (5)	0.0391 (6)	0.0122 (4)	0.0127 (5)	0.0077 (4)
C9	0.0386 (6)	0.0303 (5)	0.0221 (5)	0.0029 (4)	0.0039 (4)	0.0043 (4)
C10	0.0160 (4)	0.0183 (4)	0.0183 (4)	0.0020 (3)	0.0026 (3)	0.0012 (3)
C11	0.0206 (4)	0.0219 (4)	0.0245 (4)	0.0020 (3)	0.0050 (3)	−0.0034 (4)
C12	0.0204 (4)	0.0289 (5)	0.0290 (5)	0.0042 (4)	0.0082 (4)	−0.0045 (4)
C13	0.0171 (4)	0.0284 (5)	0.0311 (5)	0.0005 (4)	0.0070 (4)	−0.0009 (4)
C14	0.0191 (4)	0.0200 (4)	0.0273 (5)	−0.0004 (3)	0.0049 (4)	−0.0002 (4)
C15	0.0179 (4)	0.0176 (4)	0.0189 (4)	0.0032 (3)	0.0037 (3)	0.0019 (3)
C16	0.0212 (4)	0.0182 (4)	0.0225 (4)	0.0028 (3)	0.0049 (3)	−0.0022 (3)
C17	0.0290 (5)	0.0216 (5)	0.0269 (5)	0.0051 (4)	0.0016 (4)	0.0029 (4)
C18	0.0282 (5)	0.0293 (5)	0.0269 (5)	0.0045 (4)	−0.0038 (4)	0.0005 (4)
C19	0.0240 (5)	0.0248 (5)	0.0285 (5)	−0.0003 (4)	0.0005 (4)	−0.0042 (4)
C20	0.0259 (5)	0.0190 (4)	0.0270 (5)	0.0005 (4)	0.0036 (4)	0.0005 (4)
C21	0.0225 (4)	0.0207 (4)	0.0219 (4)	0.0017 (3)	0.0023 (3)	−0.0002 (3)
C22	0.0176 (4)	0.0168 (4)	0.0348 (5)	0.0006 (3)	0.0044 (4)	−0.0001 (4)
C23	0.0213 (5)	0.0234 (5)	0.0499 (7)	0.0020 (4)	0.0068 (4)	−0.0078 (5)
C24	0.0239 (5)	0.0244 (5)	0.0795 (10)	0.0079 (4)	0.0104 (6)	0.0029 (6)
C25	0.0319 (6)	0.0448 (7)	0.0752 (10)	0.0170 (6)	0.0103 (6)	0.0294 (7)
C26	0.0389 (7)	0.0583 (9)	0.0474 (8)	0.0186 (6)	0.0084 (6)	0.0263 (7)
C27	0.0274 (5)	0.0338 (6)	0.0356 (6)	0.0103 (4)	0.0077 (4)	0.0098 (5)
N1	0.0792 (9)	0.0311 (5)	0.0293 (5)	−0.0018 (5)	0.0048 (5)	−0.0086 (4)
P1	0.01578 (11)	0.01752 (11)	0.01963 (12)	0.00196 (8)	0.00391 (8)	−0.00018 (8)
P2	0.02038 (12)	0.01672 (12)	0.02347 (12)	0.00125 (9)	0.00671 (9)	−0.00103 (9)

### {2-[tert-Butyl(pyridin-2-yl)phosphino]phenyl}diphenylphosphine Geometric parameters (Å, º)

**Table:**

C1—N1	1.3299 (15)	C12—H12	0.9500
C1—C2	1.3876 (16)	C13—C14	1.3891 (14)
C1—P1	1.8461 (10)	C13—H13	0.9500
C2—C3	1.3873 (17)	C14—C15	1.4014 (13)
C2—H2	0.9500	C14—H14	0.9500
C3—C4	1.373 (2)	C15—P2	1.8469 (10)
C3—H3	0.9500	C16—C21	1.3969 (14)
C4—C5	1.368 (2)	C16—C17	1.3979 (14)
C4—H4	0.9500	C16—P2	1.8349 (10)
C5—N1	1.3432 (18)	C17—C18	1.3904 (15)
C5—H5	0.9500	C17—H17	0.9500
C6—C8	1.5291 (14)	C18—C19	1.3864 (16)
C6—C7	1.5358 (15)	C18—H18	0.9500
C6—C9	1.5359 (15)	C19—C20	1.3855 (15)
C6—P1	1.8756 (10)	C19—H19	0.9500
C7—H7A	0.9800	C20—C21	1.3906 (14)
C7—H7B	0.9800	C20—H20	0.9500
C7—H7C	0.9800	C21—H21	0.9500
C8—H8A	0.9800	C22—C27	1.3857 (16)
C8—H8B	0.9800	C22—C23	1.3995 (14)
C8—H8C	0.9800	C22—P2	1.8295 (11)
C9—H9A	0.9800	C23—C24	1.3914 (18)
C9—H9B	0.9800	C23—H23	0.9500
C9—H9C	0.9800	C24—C25	1.375 (2)
C10—C11	1.4000 (13)	C24—H24	0.9500
C10—C15	1.4105 (13)	C25—C26	1.383 (2)
C10—P1	1.8436 (9)	C25—H25	0.9500
C11—C12	1.3895 (14)	C26—C27	1.3928 (16)
C11—H11	0.9500	C26—H26	0.9500
C12—C13	1.3860 (15)	C27—H27	0.9500
			
N1—C1—C2	121.73 (10)	C14—C13—H13	120.1
N1—C1—P1	121.06 (9)	C13—C14—C15	121.38 (9)
C2—C1—P1	117.09 (8)	C13—C14—H14	119.3
C3—C2—C1	119.56 (13)	C15—C14—H14	119.3
C3—C2—H2	120.2	C14—C15—C10	118.75 (8)
C1—C2—H2	120.2	C14—C15—P2	122.13 (7)
C4—C3—C2	118.84 (13)	C10—C15—P2	118.97 (7)
C4—C3—H3	120.6	C21—C16—C17	118.46 (9)
C2—C3—H3	120.6	C21—C16—P2	124.21 (8)
C5—C4—C3	117.68 (12)	C17—C16—P2	117.33 (8)
C5—C4—H4	121.2	C18—C17—C16	120.69 (10)
C3—C4—H4	121.2	C18—C17—H17	119.7
N1—C5—C4	124.73 (15)	C16—C17—H17	119.7
N1—C5—H5	117.6	C19—C18—C17	120.16 (10)
C4—C5—H5	117.6	C19—C18—H18	119.9
C8—C6—C7	110.00 (9)	C17—C18—H18	119.9
C8—C6—C9	109.28 (9)	C20—C19—C18	119.76 (10)
C7—C6—C9	108.63 (9)	C20—C19—H19	120.1
C8—C6—P1	117.08 (7)	C18—C19—H19	120.1
C7—C6—P1	106.73 (7)	C19—C20—C21	120.23 (10)
C9—C6—P1	104.75 (7)	C19—C20—H20	119.9
C6—C7—H7A	109.5	C21—C20—H20	119.9
C6—C7—H7B	109.5	C20—C21—C16	120.66 (9)
H7A—C7—H7B	109.5	C20—C21—H21	119.7
C6—C7—H7C	109.5	C16—C21—H21	119.7
H7A—C7—H7C	109.5	C27—C22—C23	118.65 (10)
H7B—C7—H7C	109.5	C27—C22—P2	124.46 (8)
C6—C8—H8A	109.5	C23—C22—P2	116.88 (9)
C6—C8—H8B	109.5	C24—C23—C22	120.26 (12)
H8A—C8—H8B	109.5	C24—C23—H23	119.9
C6—C8—H8C	109.5	C22—C23—H23	119.9
H8A—C8—H8C	109.5	C25—C24—C23	120.33 (12)
H8B—C8—H8C	109.5	C25—C24—H24	119.8
C6—C9—H9A	109.5	C23—C24—H24	119.8
C6—C9—H9B	109.5	C24—C25—C26	120.07 (12)
H9A—C9—H9B	109.5	C24—C25—H25	120.0
C6—C9—H9C	109.5	C26—C25—H25	120.0
H9A—C9—H9C	109.5	C25—C26—C27	119.83 (14)
H9B—C9—H9C	109.5	C25—C26—H26	120.1
C11—C10—C15	119.04 (8)	C27—C26—H26	120.1
C11—C10—P1	121.36 (7)	C22—C27—C26	120.84 (11)
C15—C10—P1	119.58 (7)	C22—C27—H27	119.6
C12—C11—C10	121.32 (9)	C26—C27—H27	119.6
C12—C11—H11	119.3	C1—N1—C5	117.39 (13)
C10—C11—H11	119.3	C10—P1—C1	98.68 (4)
C13—C12—C11	119.68 (9)	C10—P1—C6	104.17 (4)
C13—C12—H12	120.2	C1—P1—C6	106.31 (5)
C11—C12—H12	120.2	C22—P2—C16	100.00 (4)
C12—C13—C14	119.79 (9)	C22—P2—C15	102.38 (5)
C12—C13—H13	120.1	C16—P2—C15	100.61 (4)
			
N1—C1—C2—C3	−2.81 (19)	C25—C26—C27—C22	1.1 (2)
P1—C1—C2—C3	−178.86 (10)	C2—C1—N1—C5	1.1 (2)
C1—C2—C3—C4	2.2 (2)	P1—C1—N1—C5	177.03 (14)
C2—C3—C4—C5	0.0 (2)	C4—C5—N1—C1	1.2 (3)
C3—C4—C5—N1	−1.7 (3)	C11—C10—P1—C1	−38.87 (9)
C15—C10—C11—C12	1.31 (15)	C15—C10—P1—C1	139.35 (8)
P1—C10—C11—C12	179.54 (8)	C11—C10—P1—C6	70.49 (9)
C10—C11—C12—C13	−2.04 (16)	C15—C10—P1—C6	−111.29 (8)
C11—C12—C13—C14	0.97 (16)	N1—C1—P1—C10	108.10 (11)
C12—C13—C14—C15	0.82 (16)	C2—C1—P1—C10	−75.83 (9)
C13—C14—C15—C10	−1.53 (15)	N1—C1—P1—C6	0.48 (11)
C13—C14—C15—P2	174.09 (8)	C2—C1—P1—C6	176.56 (8)
C11—C10—C15—C14	0.46 (14)	C8—C6—P1—C10	−46.32 (9)
P1—C10—C15—C14	−177.80 (7)	C7—C6—P1—C10	−170.02 (7)
C11—C10—C15—P2	−175.29 (7)	C9—C6—P1—C10	74.89 (8)
P1—C10—C15—P2	6.44 (10)	C8—C6—P1—C1	57.33 (9)
C21—C16—C17—C18	−2.10 (15)	C7—C6—P1—C1	−66.37 (8)
P2—C16—C17—C18	178.12 (8)	C9—C6—P1—C1	178.54 (7)
C16—C17—C18—C19	1.18 (17)	C27—C22—P2—C16	100.31 (10)
C17—C18—C19—C20	0.49 (17)	C23—C22—P2—C16	−78.92 (8)
C18—C19—C20—C21	−1.20 (16)	C27—C22—P2—C15	−3.00 (10)
C19—C20—C21—C16	0.24 (16)	C23—C22—P2—C15	177.77 (8)
C17—C16—C21—C20	1.39 (15)	C21—C16—P2—C22	−14.73 (9)
P2—C16—C21—C20	−178.85 (8)	C17—C16—P2—C22	165.04 (8)
C27—C22—C23—C24	−0.84 (16)	C21—C16—P2—C15	90.02 (9)
P2—C22—C23—C24	178.44 (9)	C17—C16—P2—C15	−90.22 (8)
C22—C23—C24—C25	1.53 (18)	C14—C15—P2—C22	100.67 (8)
C23—C24—C25—C26	−0.9 (2)	C10—C15—P2—C22	−83.73 (8)
C24—C25—C26—C27	−0.5 (2)	C14—C15—P2—C16	−2.16 (9)
C23—C22—C27—C26	−0.49 (18)	C10—C15—P2—C16	173.44 (7)
P2—C22—C27—C26	−179.71 (10)		

### {2-[tert-Butyl(pyridin-2-yl)phosphino]phenyl}diphenylphosphine Hydrogen-bond geometry (Å, º)

*Cg*2, *Cg*3 and *Cg*4 are the centroids of the C10–C15, C16–C21 and C22–C27 rings, respectively.

**Table:**

*D*—H···*A*	*D*—H	H···*A*	*D*···*A*	*D*—H···*A*
C7—H7*A*···N1	0.98	2.51	3.1946 (18)	127
C8—H8*B*···N1	0.98	2.48	3.1921 (19)	129
C9—H9*B*···*Cg*3^i^	0.98	2.97	3.8163 (14)	145
C13—H13···*Cg*4^ii^	0.95	2.85	3.6835 (12)	146
C14—H14···*Cg*3	0.95	2.97	3.7522 (12)	140
C18—H18···*Cg*2^iii^	0.95	2.71	3.6314 (12)	163

Symmetry codes: (i) −*x*+1, −*y*+1, −*z*+2; (ii) *x*+1, *y*, *z*; (iii) −*x*+2, −*y*+1, −*z*+2.
